# New Appliances and Things Medical

**Published:** 1902-01-25

**Authors:** 


### NEW APPLIANCES AND THINGS MEDICAL.

[We shall be glad to receive, at our Office, 28 & 29 Southampton Street, Strand, London, W.C., from the manufacturers, specimens of all new preparations and appliances which may be brought out from time to time.]

### FAROLA.

(James Marshall, Ltd., 25 East Cumberland Street, Glasgow.)

Farola is in character a finely divided semolina, which is prepared by a special process from pure wheat. It consists to the extent of 78 per cent. of starchy matter, and to the extent of 7 per cent. of albuminoids. The nitrogenous portions of Farola are presented in a form which is more readily digestible than that of ordinary flour. When prepared with milk according to any one of the numerous receipts recommended by the manufacturer, it constitutes an ideal food for invalids and children, since it contains all the food elements which are necessary for the maintenance of the physiological activities, and at the same time offers every facility for complete digestion and assimilation. Farola is delicious in flavour, and may be employed with the greatest advantage for thickening soup or broths, for making every variety of gruel, pudding, cake, or in a great number of other ways which are described in a very useful little brochure which is published by the manufacturers.

